# Prevalence and factors associated with unplanned pregnancy in The Gambia: findings from 2018 population-based survey

**DOI:** 10.1186/s12884-021-04371-7

**Published:** 2022-01-06

**Authors:** Amadou Barrow, Amienatta Jobe, Sulayman Barrow, Ebrima Touray, Michael Ekholuenetale

**Affiliations:** 1 Department of Public & Environmental Health, School of Medicine & Allied Health Sciences, University of The Gambia, Kanifing, The Gambia; 2 Department of Obstetrics and Gynaecology, Edward Francis Small Teaching Hospital, Banjul, The Gambia; 3 Public Health Unit, Brikama District Hospital, Ministry of Health, West Coast Region, Brikama, The Gambia; 4 School of Public Health, Gambia College, West Coast Region, Brikama, The Gambia; 5 Department of Epidemiology and Medical Statistics, Faculty of Public Health, College of Medicine, University of Ibadan, Ibadan, Nigeria

**Keywords:** Family planning, Pregnancy, Abortion, Unintended, Unwanted, Untimed, Gambia

## Abstract

**Background:**

Unplanned pregnancy is a public health issue that has detrimental implications for the mother and baby alike. However, few studies have been conducted in The Gambia on this subject. As a result, the prevalence of unplanned pregnancy among women of reproductive age in The Gambia was investigated, as well as the factors associated with it.

**Methods:**

The Gambia's Multiple Indicators Cluster Survey (MICS) was used to evaluate the 2018 results. Data was obtained from 3790 women aged 15 to 49 who had also given birth. The univariate analysis was conducted using percentage. The adjusted odds ratios (AOR) were determined using a multivariable logistic regression model (with corresponding 95% confidence interval (CI)). The degree of statistical significance was set at 5%.

**Results:**

Approximately 25.3% (95% CI: 23.1%-27.6%) of the women reported unplanned pregnancy. Women aged 30–34 years had 45% reduction in unplanned pregnancy, when compared with those aged 15–19 years (AOR = 0.55; 95% CI: 0.32–0.94). The Fula and non-Gambian women had 30% and 45% reduction in unplanned pregnancy respectively, when compared with Mandinka women. Those who had no functional difficulties had 47% reduction in unplanned pregnancy, when compared with women who had functional difficulties (AOR = 0.53; 95% CI: 0.30, 0.91). Respondents who had given births to 3–4 and 5 + children were 1.79 and 3.02 times as likely to have unplanned pregnancy, when compared with women who had given birth to 1–2 children. Single/unmarried women were 11.38 times as likely to have unplanned pregnancy, when compared with women currently married/in union (AOR = 11.38; 95% CI: 6.38, 20.29). Local Government Area of residence was significantly associated with unplanned pregnancy. Furthermore, women who were neither happy nor unhappy and 18 + at sexual debut were 1.39 and 1.34 times as likely to have unplanned pregnancy, when compared with the very happy women and those < 18 at sexual debut respectively.

**Conclusion:**

The rate of unintended pregnancies was large (25.3%). Several causes have been linked to unplanned pregnancies. These results suggest that further efforts are required to enhance women's sex education, expand access to family planning services, and provide affordable health care to high-risk women in order to minimize unintended pregnancies.

## Background

Unplanned pregnancy is a major public, clinical, and social health problem around the world because it often leads to induced abortion and subsequent complications, which are often caused by insufficient abortion care services, particularly in resource-constrained settings [1]. Between 2015 and 2019, there were 121 million unintended pregnancies, leading to a global average of 64 unintended pregnancies per 1000 women aged 15 to 49 years [2]. About 61% of unintended pregnancies resulted in abortion, resulting in a global abortion rate of 39 abortions per 1,000 women aged 15 to 49 [2]. Unintended pregnancies account for around 44% of all pregnancies globally, with around 55% of unintended pregnancies in developed countries ending in abortion [3]. In sub-Saharan Africa (SSA), there was 33.9% unintended pregnancy rate [4]. The rate of unintended pregnancies and their effects can be minimized by increasing the availability, accessibility and affordability of contraception [5]. Individuals are increasingly trying to avoid unwanted births due to long-term global decreases in ideal family size and changes in the age at which people want to start families [6].

Unplanned pregnancy can be attributed to non-use of modern contraceptive methods among sexually active women [7]. More than 200 million women in developing countries choose to postpone their next pregnancy or stop childbirth altogether, but many still rely on inadequate or obsolete contraceptive methods, if they use contraception at all [8]. According to a previous report, 342 203 women died from maternal causes, but contraceptive use avoided 272 040 maternal deaths (44%), meaning that the number of maternal deaths would have been 1.8 times higher if contraceptive use had not been used [9]. A further 104 000 maternal deaths per year could be avoided by addressing the unmet need for contraceptives (29% reduction) [9]. In several resource-poor countries, the number of unintended pregnancies and unmet contraception needs remains high. Evidence-based research has shown that the use of contraception to avoid unplanned pregnancy is a substantial and successful primary prevention method [10, 11].

If family planning needs are met to delay, space, and limit the number of children women have, millions of girls and women in the world's poorest countries, including The Gambia, will see an improvement in their living conditions. Albeit, nearly 222 million women in developing countries do not use a modern form of contraception to prevent pregnancy [12, 13]. The third Sustainable Development Goal (SDG-3) prioritizes universal access to sexual and reproductive health care services, including reducing unmet contraceptive needs. The third Sustainable Development Goal (SDG-3) aims to ensure healthier lives and foster well-being for everyone at all ages [14]. To achieve this, adequate and long-term efforts are needed to eliminate several barriers to health-care service use, including family planning, especially in resource-constrained settings.

Evidence-based studies have reported about 18.4% prevalence of unplanned pregnancy among grand multiparous Gambian women [15] and 15.3% of unintended pregnancy among women aged 15–49 years in The Gambia [16]. While substantial resources have been allocated in Gambia to the provision of modern contraceptive methods, the total fertility rate is still high at 5.4 births per woman and an annual growth rate of 3.3 percent [17]. In all health centres in the region, the Gambian National Reproductive Health Policy (NRHP) provides for the provision of free family planning services. Despite this massive investment by the government and foreign donors in family planning, the contraceptive prevalence remains low (30.4%) [18], with about 17.6% of women having unmet family planning needs [19]. According to The Gambia Bureau of Statistics, births could be wanted at the time (planned), wanted at a later time (mistimed), or not wanted at all (unwanted) [17].

Unplanned pregnancy has been associated with many factors, including socio-demographic and economic factors, early sexual debut, accessibility to health care services, unmet need for family planning, higher parity, failure of contraceptives, preference of the partner for children, and domestic abuse [20–23]. Furthermore, age and age at sexual debut, religion, marital status and parity, education, residence have been reported as determinants of unplanned pregnancy [4]. To the best of our knowledge, there is no study in the Gambia that has examined the patterns and determinants of unplanned pregnancy among Gambian women of childbearing age. The objective of this study was to examine the prevalence and factors associated with unplanned pregnancy among women of reproductive age in The Gambia.

## Methods

### Data extraction

The study relied on secondary data from the Gambia's 2018 Multiple Indicator Cluster Survey (MICS). The study's data came from 3790 women of reproductive age who had recently given birth. The Gambia MICS, which took place in 2018, provided technical guidance on the quality or excellence of survey information to be collected, statistical monitoring, data collection, and data analysis, with the goal of improving the country's national statistical capability. MICS ensures the measurement of key points on which countries depend to generate data used in policy formulations and program planning in order to track their progress toward achieving the SDGs, as well as Gambia's National Development Plan (NDP) and other international obligations to which the country is a signatory. The MICS' contribution to improving Gambian data and device control, as well as providing technical expertise in the design, implementation, and analysis of such systems. The creation of the MICS program by the United Nations International Children's Emergency Fund (UNICEF) was largely motivated by the need to assist countries in gathering data on a global scale.

The Gambia MICS 2018 sample was designed to provide estimates for a large number of indicators on the situation of children and women at the national level, for urban and rural areas, and for the eight LGAs: Banjul, Kanifing, Brikama, Mansakonko, Kerewan, Kuntaur, Janjanbureh, and Basse. A multistage stratified cluster randomized sampling was used in the survey. The first stage involved randomly selecting enumeration areas (EAs) or clusters within each substratum with a probability proportional to size. The main sampling strata within each local government area were identified as urban and rural areas, and the sample of households was selected in two stages. The primary sampling unit was household. Following the completion of a household listing within the selected enumeration areas, a systematic sample of 20 households was drawn in each sample enumeration area. During the fieldwork period, all enumeration areas were visited. The global MICS program's procedures and standard guidelines were adapted to The Gambia MICS 2018 final questionnaires and used throughout the survey.

## Variable selection and measurement

### Outcome

The study outcome was unplanned pregnancy. The outcome variable was derived from the question; “*Wanted last child then*?” This was coded as “1” if a respondent answered “no” for unplanned pregnancy and “0” if otherwise. According to The Gambia Bureau of Statistics (GBS), births wanted at the time is regarded as planned pregnancy [17].

### Explanatory variables

Table 1 showed the explanatory variables used in this study. The list of variables include: age (in years), household wealth quintiles, education, health insurance, ethnicity, functional disabilities, parity, marital status, age at first marriage/union, Local Government Area (LGA), residential status, overall happiness, age (in years) at sexual debut, ever used any method to avoid pregnancy, reading newspaper/magazine, listening to radio, watching television and family motility. Household wealth index quintiles was computed using a set of household assets in line with previous studies [24, 25]. See Table 1 for the details.

**Table 1:** Description/definition of explanatory variables

S/N	Variable	Description/definition
1	Age (in years)	15–19, 20–24, 25–29, 30–34, 35–39, 40–44, 45–49
2	Household wealth quintiles	poorest, poorer, middle, richer, richest
3	Education	pre-primary or none, primary, secondary/higher
4	Health insurance	With insurance versus without insurance
5	Ethnicity	Mandinka, Wollof, Fula, Jola, Sarahule, other ethnic groups and non-Gambians
6	Functional disabilities	has functional difficulties versus has no functional difficulties
7	Parity	1–2, 3–4, 5 +
8	Marital status	currently married/in union, formerly married/in union, single
9	Age at first marriage/union	< 18, 18–24, 25 + , never married
10	Local Government Area (LGA)	Banjul, Kanifing, Brikama, Mansakonko, Kerewan, Kuntaur, Janjanbureh, Basse
11	Residential status	urban versus rural
12	Overall happiness	very happy, somewhat happy, neither happy nor unhappy, somewhat unhappy, very unhappy
13	Age (in years) at sexual debut	< 18, 18 + , at first marriage
14	Ever used any method to avoid pregnancy	yes versus no
15	Reading newspaper/magazine	yes versus no
16	Listening to radio	yes versus no
17	Watching television	yes versus no
18	Family motility	native (lived 5 + years in the location) versus visitor (lived < 5 years in the location)

### Ethical consideration

We used publicly available datasets that were already accepted in March 2017 by the Government of Gambia and the Scientific Coordinating Committee (SCC) of the Medical Research Council (MRC) for the survey protocol. It is also on record that each survey participant received verbal consent. The ability to participate in the survey and their confidentiality and anonymity were guaranteed for all the survey respondents.

### Statistical analysis

Stata survey (‘svy’) module was used to adjust for stratification, clustering and sampling weights. The Variance Inflation Factor (VIF) was used to assess multicollinearity, using a cut-off of 10 to detect significant concerns [26]. There was no variable omitted in the model due to lack of multicollinearity. Weighted percentages were used to describe the demographic characteristics.. To estimate the adjusted odds ratios, all statistically significant variables from the bivariate analysis were included in the multivariable logistic regression model using a 20% significance level. The predictive margins was conducted as a post-estimation of logit model. The model is presented thus;

$$\mathrm{Pr}\left(Y=1|\mathrm{Set}\left[\mathrm{E}=\mathrm{e}\right]\right)=\sum_{z}{\widehat{p}}_{ez}\mathrm{Pr}\left(Z=z\right);$$

where Set[E = e] reflects putting all observations to a single exposure level e, and Z = z refers to a given set of observed values for the covariate vector Z. Furthermore, ${\widehat{p}}_{ez}$is the predicted probabilities of unplanned pregnancy (outcome variable) for any E = e and Z = z. The margin estimates indicate a weighted average over the distribution of the covariates and are equal to estimates got by standardizing to the entire population. As a post logit test, the exposure *E* is set to the level *e* for all women in the dataset, and the logit coefficients are used to compute predicted probabilities for every woman at their observed covariate pattern and newly exposure value [27, 28]. At *p* < 0.05, the statistical significance was determined in the adjusted model. For data processing, Version 14 of Stata (StataCorp., College Station, TX, USA) was used.

## Results

Overall, approximately 25.3% (95% CI: 23.1%-27.6%) of the women reported unplanned pregnancy. Table 2 showed the results of unplanned pregnancy among the Gambian women. In addition, there were differences in unplanned pregnancy across the levels of women characteristics. Unplanned pregnancy was higher among advanced age women. Women aged 40–44 and 45–49 years had 30.1% and 40.0% unplanned pregnancies respectively. Those without health insurance, from Jola ethnic group, have functional disabilities, 5 + number of children ever born and single/never married had 25.6%, 37.2%, 42.3%, 29.5%, 77.8% unplanned pregnancy respectively. See Table 2 below for the details.

**Table 2:** Distribution of unplanned pregnancy across women’s characteristics (*n* = 3,790)

Variable	Unweighted number of women	Weighted percentage of women; 95% CI	Weighted percentage of unplanned pregnancy; 95% CI
**Age**			
15–19	294	5.6 (5.1–6.3)	23.7 (16.9–32.2)
20–24	941	17.2 (16.2–18.3)	28.7 (24.2–33.8)
25–29	990	20.9 (19.7–22.1)	22.1 (18.3–26.5)
30–34	821	20.0 (18.9–21.1)	21.9 (18.3–26.0)
35–39	523	17.1 (16.2–18.1)	28.8 (23.7–34.6)
40–44	178	11.3 (10.5–12.1)	30.1 (22.0–39.6)
45–49	43	7.8 (7.2–8.5)	40.0 (22.1–61.0)
**Wealth index quintiles**			
Poorest	1362	19.4 (17.3–21.7)	25.1 (21.9–28.7)
Second	885	19.3 (17.0–21.8)	29.6 (24.8–34.8)
Middle	683	19.7 (17.4–22.2)	25.8 (21.3–31.0)
Fourth	470	21.0 (18.4–23.9)	25.1 (20.2–30.7)
Richest	390	20.6 (18.1–23.5)	19.2 (14.7–24.5)
**Education**			
Pre-primary or none	2083	47.8 (45.6–49.9)	23.7 (21.0–26.7)
Primary	706	17.0 (15.8–18.2)	24.4 (19.8–29.6)
Secondary +	1001	35.3 (33.3–37.3)	27.9 (24.5–31.7)
**Health insurance**			
With insurance	37	2.4 (1.9–3.0)	7.3 (2.3–20.9)
Without insurance	3749	97.4 (97.0–98.1)	25.6 (23.4–27.9)
**Ethnicity**			
Mandinka	1074	30.0 (27.0–33.2)	27.8 (23.9–32.1)
Wollof	694	12.6 (10.7–14.8)	26.5 (21.7–32.0)
Fula	939	20.9 (18.3–23.8)	21.1 (16.7–26.2)
Jola	157	11.1 (9.0–13.5)	37.2 (29.9–45.2)
Sarahule	402	9.1 (6.6–12.5)	11.6 (8.7–15.5)
Other	246	7.7 (6.5–9.2)	33.4 (26.2–41.4)
Non Gambian	278	8.6 (7.6–9.6)	19.3 (13.4–27.0)
**Functional difficulties**			
Has functional difficulties	86	2.2 (1.9–2.6)	42.3 (29.1–56.6)
Has no functional difficulties	3648	97.8 (97.4–98.1)	25.0 (22.8–27.4)
**Parity**			
1–2	1399	31.8 (9.1–11.0)	24.2 (20.9–27.7)
3–4	1141	26.3 (25.2–27.5)	22.3 (18.9–26.2)
5 +	1250	32.0 (30.4–33.4)	29.5 (26.3–33.0)
**Marital status**			
Currently married/in union	3611	87.0 (85.7–88.1)	22.9 (20.8–25.2)
Formerly married/in union	83	7.3 (6.6–8.1)	25.5 (15.4–39.2)
Single	72	5.7 (5.0–6.6)	77.8 (66.8–85.9)
**Age at first marriage**			
< 18	1744	40.7 (38.9–42.5)	22.3 (19.5–25.4)
18–24	1632	42.9 (41.3–44.5)	24.0 (21.3–26.9)
25 +	318	10.7 (9.8–11.6)	21.1 (16.0–27.3)
Never married	96	5.7 (5.0–6.6)	78.1 (67.1–86.1)
**LGA**			
Banjul	186	1.3 (1.1–1.4)	30.5 (22.6–39.6)
Kanifing	350	20.9 (19.3–22.7)	18.8 (15.0–23.4)
Brikama	485	38.8 (36.6–41.2)	32.4 (27.8–37.3)
Mansakonko	414	4.0 (3.5–4.5)	28.7 (24.6–33.1)
Kerewan	514	10.3 (9.2–11.5)	33.3 (29.2–37.6)
Kuntaur	617	4.8 (4.3–5.3)	19.0 (14.7–24.2)
Janjanbureh	517	6.7 (5.6–7.9)	14.6 (10.7–19.6)
Basse	707	13.3 (11.3–15.6)	13.8 (11.0–17.2)
**Residential status**			
Urban	1410	68.1 (66.1–70.1)	26.5 (23.4–29.9)
Rural	2380	31.9 (29.9–33.9)	23.2 (20.8–25.7)
**Overall happiness**			
Very happy	1551	37.2 (35.4–39.1)	22.2 (19.3–25.5)
Somewhat happy	1332	35.7 (34.2–37.1)	27.3 (24.0–30.9)
Neither happy nor unhappy	721	21.6 (20.5–22.7)	25.7 (21.7–30.2)
Somewhat unhappy	132	4.2 (3.7–4.9)	32.2 (22.6–43.7)
Very unhappy	51	1.3 (1.1–1.7)	37.9 (22.5–56.1)
**Sexual debut**			
< 18	1064	24.6 (23.2–26.0)	27.0 (22.8–31.6)
18 +	901	27.4 (25.8–29.1)	27.3 (23.9–30.9)
At first marriage	1825	48.0 (45.9–50.1)	23.4 (20.4–26.7)
**Ever used any method to avoid pregnancy**			
Yes	468	20.7 (19.3–22.1)	28.1 (22.8–34.1)
No	2633	79.3 (77.9–80.7)	23.5 (20.8–26.4)
**Reading newspaper or magazine**			
No	3584	89.1 (87.9–90.3)	25.3 (23.0–27.7)
Yes	206	10.9 (9.7–12.1)	24.6 (18.1–32.6)
**Listening to radio**			
No	783	18.4 (17.0–19.9)	21.3 (17.3–25.9)
Yes	3007	81.6 (80.0–83.0)	26.2 (23.8–28.8)
**Watching television**			
No	1733	32.0 (29.2–35.0)	24.2 (20.6–28.3)
Yes	2055	68.0 (65.0–70.8)	25.9 (23.4–28.6)
**Mobility**			
Native (lived 5 + years in the location)	2804	70.4 (68.6–72.1)	26.5 (24.1–29.1)
Visitor (lived < 5 years in the location)	986	29.6 (27.9–31.4)	22.6 (23.1–27.6)

In Table 3, we showed the factors associated with unplanned pregnancy among Gambian women. Women aged 30–34 years had 45% reduction in unplanned pregnancy, when compared with those aged 15–19 years (AOR = 0.55; 95% CI: 0.32, 0.94). The Fula and non-Gambian women had 30% (AOR = 0.70; 95% CI: 0.54, 0.92) and 45% (AOR = 0.55; 95% CI: 0.36, 0.86) reduction in unplanned pregnancy respectively, when compared with the Mandinka women. Those who had no functional difficulties had 47% reduction in unplanned pregnancy, when compared with women who had functional difficulties (AOR = 0.53; 95% CI: 0.30, 0.91). Respondents who had given births to 3–4 and 5 + children were 1.79 (AOR = 1.79; 95% CI: 1.34, 2.39) and 3.02 (AOR = 3.02; 95% CI: 2.08, 4.39) times as likely to have unplanned pregnancy respectively, when compared with women who had given birth to 1–2 children. Single women were 11.38 times as likely to have unplanned pregnancy, when compared with women currently married/in union (AOR = 11.38; 95% CI: 6.38, 20.29). LGA was significantly associated with unplanned pregnancy. Furthermore, women who were neither happy nor unhappy and 18 + at sexual debut were 1.39 and 1.34 times as likely to have unplanned pregnancy, when compared with the very happy women and those < 18 at sexual debut. See Table 3 for the details.

**Table 3:** Factors associated with unplanned pregnancy in The Gambia

Variable	Unadjusted odds ratio (95% CI)	P	Adjusted odds ratio (95% CI)	P
**Age**		< 0.001*		
15–19	1.00		1.00	
20–24	1.00 (0.81–1.23)		0.89 (0.57–1.39)	0.607
25–29	0.90 (0.73–1.11)		0.62 (0.38–1.01)	0.056
30–34	1.07 (0.86–1.32)		0.55 (0.32–0.94)	0.029*
35–39	1.41 (1.13–1.76)		0.66 (0.37–1.18)	0.158
40–44	1.90 (1.45–2.50)		0.68 (0.35–1.34)	0.265
45–49	1.81 (1.15–2.85)		0.85 (0.34–2.17)	0.742
**Household wealth quintiles**		0.279		
Poorest	1.00			
Second	1.16 (1.02–1.1.32)			
Middle	0.95 (0.82–1.10)			
Fourth	1.16 (0.99–1.36)			
Richest	0.93 (0.77–1.11)			
**Education**		0.086*		
Pre-primary or none	1.00		1.00	
Primary	0.99 (0.86–1.13)		0.95 (0.73–1.23)	0.700
Secondary +	1.21 (1.08–1.36)		1.11 (0.85–1.44)	0.434
**Health insurance**		0.065*		
With insurance	1.00		1.00	
Without insurance	2.40 (1.22–4.75)		7.28 (0.95–55.62)	0.056
**Ethnicity**		< 0.001*		
Mandinka	1.00		1.00	
Wollof	1.05 (0.91–1.21)		1.02 (0.75–1.37)	0.921
Fula	0.64 (0.55–0.74)		0.70 (0.54–0.92)	0.010*
Jola	1.78 (1.41–2.24)		1.02 (0.64–1.62)	0.939
Sarahule	0.50 (0.41–0.62)		0.77 (0.52–1.13)	0.187
Other	1.38 (1.14–1.69)		1.14 (0.77–1.69)	0.501
Non Gambian	0.70 (0.56–0.87)		0.55 (0.36–0.86)	0.008*
**Functional difficulties**		0.004*		
Has functional difficulties	1.00		1.00	
Has no functional difficulties	0.50 (0.32–0.79)		0.53 (0.30–0.91)	0.021*
**Parity**		< 0.001*		
1–2	1.00		1.00	
3–4	1.02 (0.90–1.16)		1.79 (1.34–2.39)	< 0.001*
5 +	1.67 (1.49–1.88)		3.02 (2.08–4.39)	< 0.001*
**Marital status**		< 0.001*		
Currently married/in union	1.00		1.00	
Formerly married/in union	1.28 (0.92–1.77)		1.33 (0.74–2.40)	0.339
Single	11.80 (8.64–16.11)		11.38 (6.38–20.29)	< 0.001*
**Age at first marriage**		< 0.001*		
< 18	1.00		1.00	
18–24	1.01 (0.91–1.13)		0.96 (0.77–1.19)	0.696
25 +	1.11 (0.92–1.34)		1.23 (0.84–1.80)	0.278
Never married	12.07 (8.81–16.56)		-	
**LGA**		< 0.001*		
Banjul	1.00		1.00	
Kanifing	0.53 (0.40–0.69)		0.46 (0.27–0.78)	0.004*
Brikama	1.02 (0.81–1.30)		0.87 (0.53–1.42)	0.586
Mansakonko	0.90 (0.70–1.15)		0.66 (0.37–1.17)	0.159
Kerewan	1.12 (0.88–1.42)		0.74 (0.43–1.29)	0.292
Kuntaur	0.50 (0.39–0.63)		0.33 (0.18–0.59)	< 0.001*
Janjanbureh	0.40 (0.32–0.52)		0.27 (0.15–0.48)	< 0.001*
Basse	0.37 (0.29–0.48)		0.35 (0.20–0.62)	< 0.001*
**Residential status**		0.101*		
Urban	1.00		1.00	
Rural	0.88 (0.79–0.97)		1.35 (0.98–1.87)	0.063
**Overall happiness**		0.172*		
Very happy	1.00		1.00	
Somewhat happy	1.20 (1.07–1.35)		1.21 (0.97–1.51)	0.097
Neither happy nor unhappy	1.22 (1.06–1.40)		1.39 (1.07–1.80)	0.013*
Somewhat unhappy	1.25 (0.95–1.64)		1.24 (0.74–2.09)	0.417
Very unhappy	1.47 (0.98–2.22)		1.01 (0.45–2.29)	0.978
**Sexual debut**		0.003*		
< 18	1.00		1.00	
18 +	1.29 (1.13–1.48)		1.34 (1.01–1.77)	0.042*
At first marriage	0.94 (0.84–1.06)		1.10 (0.87–1.39)	0.430
**Ever used any method to avoid pregnancy**		0.026*		
Yes	1.00		1.00	
No	0.77 (0.66–0.89)		0.91 (0.71–1.17)	0.453
**Reading newspaper or magazine**		0.420		
No	1.00			
Yes	1.15 (0.93–1.42)			
**Listening to radio**		0.084*		
No	1.00		1.00	
Yes	1.18 (1.04–1.34)		0.97 (0.76–1.23)	0.800
**Watching television**		0.003*		
No	1.00		1.00	
Yes	1.27 (1.14–1.40)		1.20 (0.97–1.48)	0.093
**Mobility**		0.100*		
Native (lived 5 + years in the location)	1.00		1.00	
Visitor (lived < 5 years in the location)	0.86 (0.76–0.97)		1.13 (0.89–1.44)	0.329

In Table 4, the predictive margins estimates were calculated to decipher the effects of the factors associated with unplanned pregnancy. From the predictive margins results, assuming the distribution of all factors remained the same among women, but every woman was aged 15–19 years or without health insurance, we would expect 26.9% and 21.0% of unplanned pregnancy respectively. If every woman had functional difficulties, we would expect 31.6% of unplanned pregnancy. If instead the distribution of women’s age, education, health insurance, ethnicity, functional difficulties, parity, were as observed and other covariates remained the same among women, but all women lived in Banjul, we would expect 33.3% of unplanned pregnancy. See Table 4 for the details.

**Table 4:** Predictive margins of unplanned pregnancy among women of reproductive age in The Gambia

Variable	Predictive margins	95% CI
**Age**		
15–19	26.9	19.0–34.8
20–24	24.9	21.0–28.9
25–29	19.4	16.6–22.1
30–34	17.8	15.0–20.5
35–39	20.2	16.4–24.1
40–44	20.8	14.6–27.0
45–49	24.3	11.6–36.9
**Education**		
Pre-primary or none	20.6	18.7–22.5
Primary	19.9	16.7–23.0
Secondary +	22.2	19.0–25.4
**Health insurance**		
With insurance	4.1	-3.5–11.7
Without insurance	21.0	19.6–22.4
**Ethnicity**		
Mandinka	22.9	20.2–25.6
Wollof	23.1	19.3–27.1
Fula	17.7	15.0–20.5
Jola	23.2	16.1–30.3
Sarahule	19.0	14.1–23.9
Other	25.1	19.2–31.0
Non Gambian	14.8	10.3–19.3
**Functional difficulties**		
Has functional difficulties	31.6	21.2–42.0
Has no functional difficulties	20.6	19.2–22.0
**Parity**		
1–2	13.8	11.6–16.1
3–4	21.2	18.5–23.9
5 +	30.1	26.0–34.2
**LGA**		
Banjul	33.3	23.7–43.0
Kanifing	19.5	13.5–25.4
Brikama	30.6	24.4–36.8
Mansakonko	25.5	20.8–30.2
Kerewan	27.6	23.0–32.2
Kuntaur	15.1	11.8–18.5
Janjanbureh	12.7	9.4–16.0
Basse	16.0	12.6–19.3
**Residential status**		
Urban	18.2	15.3–21.1
Rural	22.6	20.2–25.0
**Overall happiness**		
Very happy	18.8	16.7–20.9
Somewhat happy	21.6	19.2–23.9
Neither happy nor unhappy	23.8	20.5–27.1
Somewhat unhappy	22.0	14.3–29.7
Very unhappy	19.0	7.9–30.1
**Sexual debut**		
< 18	19.2	16.5–21.8
18 +	23.5	20.3–26.7
At first marriage	20.5	18.5–22.5
**Ever used any method to avoid pregnancy**		
Yes	22.1	18.5–25.6
No	20.6	19.1–22.1
**Listening to radio**		
No	21.2	18.0–24.4
Yes	20.7	19.2–22.3
**Watching television**		
No	19.4	17.3–21.5
Yes	22.1	20.0–24.2
**Mobility**		
Native (lived 5 + years in the location)	20.4	18.8–22.0
Visitor (lived < 5 years in the location)	22.2	19.1–25.4

## Discussion

To the best of our knowledge, this is the first study that has examined the prevalence and factors associated with unplanned pregnancy in The Gambia using a population-based data. Based on the findings of this study, approximately one-quarters of Gambian women of reproductive age reported unplanned pregnancy. Since poor use of modern contraceptive methods is the origin of unintended pregnancy [18], the relatively high prevalence of unplanned pregnancy is expected. This may be a major contributing factor to the degree of unintended pregnancy [19]. This is in line with results from a number of recent studies that looked at the prevalence of, and/or unmet needs for contraceptive use in The Gambia [18, 19]. This is consistent with previous findings on the prevalence of unplanned or unintended pregnancy in several resource-poor settings. In SSA, there was a pooled 33.9% prevalence of unintended pregnancy [4]. In Nigeria, Kenya and Ethiopia, this was about 11.0% and 24.0% respectively [29–31]. In a multi-country study, an overall unintended pregnancy prevalence rate of 29% was found, ranging from 10.8% in Nigeria to 54.5% in Namibia [16]. Results from the 2014 data showed a prevalence of 15.3% [16], whereas using the 2018 data, this increased to one-quarters indicating an upward trend.

While the Gambian government may have recognized the issue of unplanned pregnancies and committed to improving reproductive and sexual health care services, evidence suggests that there has been insufficient progress. Several factors were identified to be associated with unplanned pregnancy. In contrast to women aged 15–19 years, women in other age groups were more likely to have unplanned pregnancies, according to our research. This is consistent with the findings from a previous study [16]. Also, this result is in line with a study by Hubacher, Mavranezouli, and McGinn [32], who examined the prevalence of unintended pregnancies in SSA and the possible role of contraceptive implants in reducing them. They discovered that women aged 20–24, 25–29, and 30–34 years had the highest proportion of unintended pregnancies, compared to those aged 15–19 years [32]. According to Calvert et al. [33], who also reported from Tanzania, the risk of unplanned pregnancies rises with age. This result may be clarified by the fact that adult women might already have the ideal number of children and thus view any additional pregnancy as undesired or unplanned. Furthermore, women who had no functional difficulties had reduction in unplanned pregnancy, when compared with those with functional difficulties. Women with functional difficulties may lack physical accessibility to sexual and reproductive health care services, such as contraceptive products and health information as access to health information has been reported to have positive impact to health-seeking behaviour [34–37].

Furthermore, unplanned pregnancies were more common among single or unmarried women. This is consistent with the findings from a previous study which indicated that increased risk of unplanned pregnancy among single women [31], could be attributed to non-use of contraception or contraceptive failure, as certain single women may assume that contraceptive use has significant side effects [38], whereas others may believe that it reduces sexual pleasure, and therefore have unprotected intercourse, raising the risk of unplanned pregnancies [22, 38–42]. In addition, higher parity was a predisposing factor of unplanned pregnancy [31]. Women with higher number of children ever born could be regarded as already having the desired number of children and thus consider any additional pregnancy as unplanned or unwanted. On the other hand, women aged 18 + at sexual debut had higher odds of unplanned pregnancy. This is contrary to a previous study which found that unplanned pregnancy was associated with younger age at sexual debut [33].

Unplanned pregnancies were found to be substantially correlated with geographic area and ethnicity [29]. We found the Fulas and non-Gambian women had reduction in unplanned pregnancy respectively, when compared with the Mandinka women. In addition, women’s LGA of origin was significantly associated with unplanned pregnancy. It is possible there are more healthcare institutions in certain geographical locations, or related interventions may have been conducted within an ethnic group or geographical locations. The geospatial differentials in health care service utilization has been reported in previous studies [43–46].

### Strengths and limitations

The strengths of this analysis are that the pregnancy aim determination was not affected by the result of pregnancy and that the probability of recall bias was minimized because the data were only obtained for the index pregnancy. The use of population-based data was useful and representative for plausible comparisons. There are still, however, some drawbacks. First, other scholars raised doubts about how planned and unplanned pregnancies are described (for example, since the reactions of a woman to a pregnancy she has categorized as unplanned may be conflicting or ambivalent), as well as about discrepancies between the purpose of a woman and her pregnancy feelings. Therefore, it may be a limitation of our research to use a single item to assess pregnancy intent. Second, we were unable to find causal links between the purpose of pregnancy and the established risk factors because of the cross-sectional study design.

## Conclusion

This research showed that the prevalence of unplanned pregnancy was relatively high (25.3%) in the study settings, indicating the need for more exposure to this issue. Family planning services for women of reproductive age, especially those at high risk of unplanned pregnancy, should be accessible widely and easily. Our findings indicate, in particular, that better sex education and increased awareness of contraceptive methods are required for women. In addition, to improve the efficacy and quality of the use of contraceptive methods, women without family support can require individualized guidance and counselling.

## Data Availability

The issue of privacy of the respondents was considered such that exclusive information such as respondents’ locations and their names as collected during the MICS interviews were consciously removed from datasets. These data files are made available on; www.gbosdata.org and on the MICS website and can be freely downloaded for legitimate research purposes.

## Abbreviations

CI: Confidence Interval
GBS: Gambia Bureau of Statistics
LGA: Local Government Area
MRC: Medical Research Council
MICS: Multiple Indicator Cluster Survey
NDP: National Development Plan
NRHP: National Reproductive Health Policy
SCC: Scientific Coordinating Committee
SSA: Sub-Saharan Africa
SDG: Sustainable Development Goal
TX: Texas
UNICEF: United Nations International Children's Emergency Fund
USA: United States of America
